# *BJPsych Bulletin* author mentoring scheme – helping trainees become published authors

**DOI:** 10.1192/pb.bp.115.053215

**Published:** 2016-02

**Authors:** Jonathan Pimm, Niall Galbraith

**Affiliations:** 1 East London NHS Foundation Trust; 2 University of Wolverhampton

## Abstract

The publishing world is changing rapidly. Innovations include the move to open access, the rise of social media and the transition to digitalisation. In the light of these developments and with ever-increasing pressures on early career psychiatrists and trainees to publish papers in journals with a recognised pedigree, the *BJPsych Bulletin* is piloting an author mentoring scheme. Mentors will help clinicians and aspiring academics develop articles from a pedestrian manuscript to one that will hopefully provoke important debate and aid changes in current practices. The scheme will run on a trial basis for approximately 12 months and will then be reviewed. Mentoring has been found to have an important effect of research output including publication and grant success; the hope is that this new initiative at the *BJPsych Bulletin* will result in such dividends to all involved.

The pace of change in scientific publishing is giddying. Fast-evolving developments include a growing pressure to publish open access, the transition to digitalisation and the rise of social media. And although industry experts are unable to predict the ultimate appearance of the academic journal landscape, they are all in agreement that it will continue to evolve for quite some time.^1^

In addition to these innovations and changes there are ever-increasing pressures on early career psychiatrists, trainees and other budding academics to publish in well-recognised journals: such an achievement might provide the necessary edge to succeed at a job interview or in a grant application. The *BJPsych Bulletin* has gained a reputation for providing a platform for nascent researchers to begin publishing their work. And so, to help scientists in the mental health field navigate the scientific publishing industry and compete in the career market, the *BJPsych Bulletin* is now piloting a mentoring scheme for authors that have submitted papers to the journal.

Mentoring has a long history as a key component providing assistance to junior staff looking for career progression. It was developed in the USA in the 1970s within large private sector organisations. Formal mentoring schemes for medical students and early career doctors began to be used in the late 1990s.^2^ Primarily, mentoring has been seen as an aide for budding academics keen to develop their research portfolio, but a systematic review of mentoring in academic medicine concluded that it was an important influence on personal development, career guidance, career choice and productivity. Furthermore, it was found that mentoring had an important effect on research output, including publication and grant success.^3^ Although the provision of hard evidence demonstrating clear links between mentoring and important outcomes is limited, surveys of mentees have found that mentored students rate their overall well-being as higher.^2^

The success of mentoring schemes depend on a variety of factors including the characteristics of the mentee (e.g. younger age), experience and skills of the mentor and the quality of the relationship between those involved.^4^ The aim of the *BJPsych Bulletin* mentoring scheme is to provide input to articles with potential that are in need of revising before reaching a sufficient standard whereby they can be sent out for review. Feedback will focus on the study design and statistical analyses as well as writing tips including how to contextualise the paper in light of the current scientific thinking, both in the UK and internationally. In addition, mentors will advise about how to maximise the article's impact through the use of search-engine friendly titles, abstracts and keywords. The remit of the papers appropriate for the *BJPsych Bulletin* will be unchanged: service provision and development, teaching and education, legal, technological and cultural advancements relating to treatment and delivery of care, case reports and miscellaneous articles on governmental policy.

Mentors will help clinicians and aspiring academics understand the difference between a solid but pedestrian manuscript and one that will provoke important debate and aid the development of current practices. Mentors will be experienced researchers or academics – in some cases members of the editorial board of the *BJPsych Bulletin* – with top-level teaching skills and bags of motivation!

The idea is that mentors and mentees will agree a predetermined timetable of tasks and activities. Although there will be no formal contract, it is important to avoid acrimonious disputes which sometimes arise if a paper undergoes multiple revisions and reviews; understandably, the more time invested and the more changes made, the more some authors become convinced of the value of their work. To minimise this possibility, the mentor will have the final word over the whole process and if at any stage it appears that the article will not reach the standard required for reviewing, it will be up to the mentor to decide when to draw stumps.

With the understanding that time is a precious commodity to all healthcare professionals, it is envisaged that the average mentoring process for a paper, from start to finish, would require a maximum of about 12 hours over a period of up to 6 months: this estimate comes from a belief that no paper needing more than six revisions each taking about 2 hours will have reached the threshold for inclusion in the scheme.

Authors wishing to have papers considered for mentoring should submit them through the website (http://submit-pb.rcpsych.org/) along with a cover letter requesting entrance into the scheme. In addition, authors of articles already submitted to the *BJPsych Bulletin* but which the Editor or a member of the Editorial Board determines would benefit from mentoring will be contacted with an offer. If the paper is eventually published, credit will be given to the mentor by an acknowledgement added to the end of the article.

The scheme will run initially on a trial basis for the first 12 months and will be subject to regular informal reviews. These evaluations will be assessed by the journal's Editorial Board at the end of the year to decide the feasibility of continuing the scheme. We are very excited about this new initiative to help trainees with publication and we invite you to take part.
